# miRNA profiling in pediatric and young adult Burkitt leukemia and lymphoma

**DOI:** 10.1007/s00428-026-04426-0

**Published:** 2026-02-06

**Authors:** Björn Schneider, Caterina Redwanz, Veronica Celis, Elena Esperanza-Cebollada, Sara Montesdeoca, Itziar Salaverria, Silvia Planas, Nuria Conde, Mireia Camós, Raquel Arnau, Armando Lopez-Guillermo, Claudia Maletzki, Cristina Jou, Andreas Erbersdobler, Olia Shokraie, Almut Meyer-Bahlburg, Manfred Ballmann, Jaume Mora, Elias Campo, Carl Friedrich Classen, Teresa M. Cardesa-Salzmann

**Affiliations:** 1 Institute of Pathology, Rostock University Medical Center, Rostock, Germany; 2 Department for Internal Medicine B, Cardiology, University Medicine Greifswald, Greifswald, Germany; 3 Pediatric Cancer Center Barcelona, Hospital Sant Joan de Déu, Barcelona, Spain; 4 Institut de Recerca Hospital Sant Joan de Déu, Developmental Tumors Biology Group, Leukemia, and Other Pediatric Hemopathies, Pediatric Cancer Center Barcelona, Barcelona, Spain; 5 Institute of Biomedical Research August Pi Sunyer, Barcelona, University of Barcelona, Barcelona, Spain; 6 Anatomical Pathology Department, Hospital Sant Joan de Déu, Barcelona, Spain; 7 Department of Medicine, Clinic III-Hematology, Oncology, Palliative Medicine, Rostock University Medical Center, Rostock, Germany; 8 Institute of Clinical Chemistry and Laboratory Medicine, Rostock University Medical Center, Rostock, Germany; 9 Pediatrics Department, Rostock University Medical Center, Rostock, Germany; 10 Pediatrics Department, Pediatric Hematology, Oncology and Palliative Care, Rostock University Medical Center, Rostock, Germany

**Keywords:** Pediatric Burkitt leukemia, Pediatric Burkitt lymphoma, MiRNAs, Clinicopathological features, Outcome

## Abstract

A translational gap exists in Burkitt leukemia (B-AL) and Burkitt lymphoma (B-Ly) regarding miRNAs associated with clinicopathological features and outcome. The aim of this study was to evaluate differential miRNA expression in a single-center series of pediatric B-AL/B-Ly. Expression profiles of 800 miRNAs in 33 B-AL/B-Ly samples were evaluated using the NanoString nCounter System. Further validation was performed by qPCR utilizing miRNA-specific TaqMan assays. Significantly expressed miRNAs in B-AL/B-Ly were evaluated in silico to identify predicted targeted cancer-related pathways. Analysis of miRNAs deregulated in B-AL/B-Ly compared to normal control lymphoid tissue (NCLT) identified a consistent set of differentially expressed miRNAs, including miR-494-3p, miR-4286, and miR-19a-3p among the higher expressed miRNAs and miR-150-5p, miR-450b-5p, and miR-342-3p among the lower expressed miRNAs in B-AL/B-Ly compared to NCLT (FC > 1.5, *p*-adj < 0.05). In silico, the main predicted cancer-related signaling pathways targeted by these miRNAs included the MAPK, PI3K-Akt, JAK-STAT, VEGF, TP53, Fas, TGF-β, and MYC signaling pathways (*p*-adj < 0.05). B-AL and B-Ly segregated into two major miRNA clusters with sets of significantly overexpressed miRNAs (miR-223-3p, miR-451a, miR-150-5p, miR-144-3p, miR-142-3p, and miR-15a-5p) and lower expressed miRNAs (miR-494-3p, miR-4286, miR-1915-3p, miR-125b-5p, and miR-100-5p) in B-AL compared to B-Ly (FC > 1.5, *p*-adj < 0.05). Notably, significant downregulation of miR-10a-5p (FC > 1.5, *p-*adj < 0.05) was observed in the unfavorable outcome group. In summary, new miRNA signatures of relevance in B-AL and B-Ly could be recognized in this study. Studies in larger cohorts are required to further validate these findings.

**Supplementary Information:**

The online version contains supplementary material available at https://doi.org/10.1007/s00428-026-04426-0.

## Introduction

Burkitt lymphoma (B-Ly) is the most common B-cell non-Hodgkin lymphoma (NHL) in children, accounting for 40% of NHL in children and 1–2% of NHL in adults [1]. First described by Dennis Burkitt in 1958 [2], B-Ly is an aggressive mature B-cell neoplasm with a B-cell phenotype resembling germinal center B (GCB) cells [3, 4], most closely the dark zone cells [5], showing CD10 +, BCL6 +, BCL2-/weak, high Ki67 index (> 95%) and an *IG*::*MYC* translocation [6]. B-Ly is characterized by dysregulation of *MYC* due to enhancer activation via juxtaposition with one of the three immunoglobulin loci [6, 7], resulting in the inappropriate expression of *MYC*. Although *MYC* translocations are present in all B-Ly, further cooperating genomic alterations appear to be required for lymphoma development [8–10]. Genomic and transcriptomic studies have identified additional genomic alterations, including *TP53* [11, 12], *ID3*, *TCF3* and *CCND3* mutations [9, 11], as well as inactivation of the tumor suppressors *ARF* [13] and *DDX3X* [14], among others. Historically the three subgroups “endemic”, “sporadic”, and “immunodeficiency-associated” were described. Recent data suggest that EBV-positive B-Ly and EBV-negative B-Ly form separate biologic subgroups [15–17] and acknowledging these insights, the distinction of EBV-positive B-Ly and EBV-negative B-Ly is recommended [6].

In B-Ly the most common primary sites of disease are the Waldeyer ring, lymph nodes of the head and neck region as well as involvement of the abdomen [10, 18]. Extranodal spread is common [10, 18]. Bone marrow (BM) infiltration below 25% occurs in 30–35% of B-Ly cases and central nervous system involvement in up to 20% of B-Ly cases [10]. Burkitt leukemia (B-AL) is defined by more than 25% BM involvement by cytomorphology, a germinal center B-cell immunophenotype by flow cytometry and an *IG::MYC* on more than 25% of malignant cells [3, 4]. B-AL can present as a leukemic disease either in combination with large tumors or without extramedullary involvement [3]. Both B-AL and B-Ly with BM involvement below 25% are treated as stage IV disease [19]. Due to overlapping clinical and biologic features, including morphology, immunophenotype, and genotype, B-AL and B-Ly are considered different presentations of a single disease. Precursor B-cell acute lymphoblastic leukemia with *IG::MYC*, which has an immature phenotype, is molecularly distinct from B-Ly and is considered a different entity [20].

MicroRNAs (miRNAs) are small, single-stranded, non-coding ribonucleic acid (RNA) molecules containing 19 to 22 nucleotides. By base-pairing to complementary sequences at the 3′-UTR of numerous target mRNAs, miRNAs lead to mRNA degradation or translational repression thereby regulating post-transcriptionally the expression of large gene networks [21]. Emerging evidence suggests that abnormal modulation of mRNA transcription via miRNAs might be a significant factor in B-Ly pathogenesis [22]. miRNAs regulate gene expression either by directly targeting specific genes or by targeting regulatory elements such as transcription factors, whose impact propagates across the whole regulatory network to contribute to B-Ly development. Differential miRNA expression patterns have been evaluated in B-Ly with regard to normal GCB-lymphocytes [23], other non-*MYC*-driven B-NHL [24–26] and B-Ly cell lines [27]. The aim of this study was to gain knowledge on differential miRNA expression in a single center series of pediatric B-AL and B-Ly. Comparison of groups based on tumor and normal control lymphoid tissue (NCLT), as well as based on clinical characteristics was performed to evaluate miRNAs deregulated in B-AL and B-Ly.

## Materials and methods

### Clinical characteristics overview

This study was approved by the Ethics Committees of the University of Rostock (Reference number: A 2023–0054) and the University of Barcelona (HSJD C.I. PIC-36–23, HCP HCB/2022/1233). Specimen collection was conducted in accordance with the ethics guidelines for the use of human material and in accordance with the Declaration of Helsinki. Informed consent was obtained from all patients or their legal guardians.

We evaluated 33 B-AL/B-Ly cases diagnosed between 2006 and 2021 in children and young adults (CAYA) at the Hospital Sant Joan de Déu of Barcelona, Spain, and now fulfilling the diagnostic criteria for B-AL/B-Ly according to the International Consensus Classification of Mature Lymphoid Neoplasms [4] and the 5th edition of the WHO-classification on mature lymphoid neoplasms [6]. All cases were *MYC* rearrangement (MYC-R) positive by fluorescent in situ hybridization (FISH). Precursor-B-cell acute lymphoblastic leukemia cases with *IG::MYC* [20] were excluded from this study. All cases were HIV negative. Cases were homogeneously treated with the LNH-B-04 protocol until 2009 and with the Inter-B-Ritux 2010 Protocol [19] starting in 2010. The median age at diagnosis was 8 years (range 3 years 4 months–18 years 11 months), 75.7% of patients were male and 24.3% were female. Median age of patients providing NCLT samples obtained with diagnostic purposes was 9 years (range 2 years 6 months–12 years 5 months). Of the 26 B-Ly cases 15.38% were stage II disease according to the St. Jude staging system, 53.8% stage III and 30.8% stage IV. B-AL was defined as more than 25% blasts in BM by cytomorphology and was confirmed by flow cytometry (range 32–100%, median 38%). Stage IV disease due to BM involvement was defined by morphologic evidence of lymphoma cells in BM aspirate below 25% and confirmed by flow cytometry (range 1–6%, median 3%). Stage IV disease due to central nervous system (CNS) involvement was defined by either evidence of blasts morphologically identified in the cerebrospinal fluid, CNS tumor mass or cranial nerve palsy not explained by extradural lesions. All cases were de novo except for one B-Ly case with relapsed disease. The clinical characteristics are summarized in Table 1.

**Table 1:** Clinical characteristics

	Burkitt leukemia (*n* = 7)	Burkitt lymphoma (*n* = 26)	Total (*n* = 33)
Median age (years) (range)	8 (5–15)	7.5 (3–18)	8 (3–18)
Pediatric (< 18y)	*N* = 7	*N* = 24	
Young adult (> = 18y)	*N* = 0	*N* = 2	
Gender (M/F)	6/1 (85.7%/14.3%)	19/7 (73.1%/26.9%)	25/8 (75.7%/24.3%)
Stage			
I		0 (0%)	
II		4 (15.4%)	
III		14 (53.8%)	
IV		8 (30.8%)	
Bone marrow involvement			
> 25%	7 (100%)		
< 25%			
Yes		3 (11.5%)	
No		23(88.4%)	
CNS involvement			
Yes	5 (71.4%)	8 (30.8%)	13 (39.4%)
No	2 (28.6%)	18 (69.2%)	20 (60.6%)
*MYC-R* (FISH)	7 (100%)	26 (100%)	33 (100%)
EBER			
Positive		5 (19.2%)	
Negative		21(80.8%)	
LNH-B-O4-Protocol	1	4	5 (15.2%)
InterB-Ritux-2010-Protocol	6	22	28 (84.8%)
Relapse			
Yes	1	1	2 (6%)
No	6	25	31 (94%)

### Tumor samples and normal control lymphoid tissue

Formalin-fixed paraffin-embedded (FFPE) sections of Burkitt lymphoma tumor tissue material were obtained from the archives of the Department of Pathology of the Hospital Sant Joan de Déu and from the Hematopathology Department of the Hospital Clinic, University of Barcelona, Barcelona, Spain. For all cases, hematoxylin and eosin-stained slides of the selected paraffin blocks were examined by experienced hematopathologists to ensure sample sufficiency and quality. All samples contained more than 60% of neoplastic cells. After flow cytometric analysis, exceeding material of B-AL bone marrow aspirate samples was used for miRNA extraction after treatment of samples with a Ficoll-Hypaque (Sigma, St Louis, MO, USA) centrifugation gradient.

After flow cytometric analysis showing no evidence of malignant cell infiltration, exceeding material from normal tonsils (*n* = 2) and a normal lymph node (*n* = 1) was used for miRNA extraction. Mixes were created for comparisons with B-AL and B-Ly (Supplementary file 1_B-AL, B-Ly, NCLT data sheet).

### miRNA extraction

For miRNA extraction from FFPE material, the miRNeasy FFPE-Kit (Qiagen, Hilden, Germany) was used as per manufacturer’s instructions. Total RNA from bone marrow mononuclear cells and from NCLT was extracted with TRIzol using the TriPure Isolation Reagent (Roche Diagnostics, Indianapolis, IN, USA) and the Direct-zol RNA MiniPrep kit (Zymo Research, California, USA). Total RNA was stored at − 80 °C with DEPC H20.

### miRNA screening arrays

Analysis of miRNA expression was performed using the NanoString nCounter System with the Human v3 miRNA assay (Nanostring, Seattle, WA, USA), analyzing 800 miRNAs per sample following the manufacturer’s instructions. 100 ng of total RNA was included per sample. Raw data were imported from the nCounter Digital Analyzer and analyzed with the Rosalind® software (https://rosalind.bio/).

### Statistical analysis

Following background subtraction based on probe correction factors, normalization was performed in two steps, including positive control and codeset normalization. Group-wise comparisons with the *t*-test method were performed, delivering fold changes and *p*-values. *P*-value adjustment was performed through the Benjamini–Hochberg method of estimating false discovery rates (FDR). Clustering of miRNAs for the final heatmap of differentially expressed miRNA was done using the PAM (Partitioning Around Medoids) method.

### miRNA validation

Four miRNAs among the most significantly up-and downregulated in B-AL compared to B-Ly were chosen for exemplary validation of miRNA array screening results by qPCR using miRNA-specific TaqMan Assays (Applied Biosystems, Darmstadt, Germany), containing RT-primers specific for the mature miRNAs. RNU6B was used as endogenous control (Applied Biosystems) in the entire cohort. The reverse transcription was carried out as a multiplex reaction, containing the RT-primers for all four miRNAs and the endogenous control in one reaction. The subsequent qPCR reactions were set up according to the manufacturer’s protocol using TaqMan Universal PCR Master Mix II, No UNG (Applied Biosystems) and the miRNA-specific primer/probe mixes in singleplex reactions. The runs were performed in triplicates on a StepOne Plus Real-time PCR system (Applied Biosystems) and the data analyzed using the StepOne Software v2.3 (Applied Biosystems). Relative expression against RNU6B (fold change) was calculated using the 2^−ΔCt^-algorithm. Statistical significance analysis of comparisons between groups was assessed through the U-Mann–Whitney test.

### Bioinformatic analysis of validated target genes and pathways

Significantly expressed miRNAs in B-AL and B-Ly were evaluated with regard to their validated target genes and involved signaling pathways through bioinformatic analysis with the Rosalind® platform using the multiMiR R library (http://multimir.ucdenver.edu) [28] and the DIANA-miRPath v4® KEGG [29–31] pathway enrichment analysis (http://www.microrna.gr/miRPathv4) [32].

## Results

The group-wise comparison of the miRNA expression profiles obtained by the NanoString nCounter assays revealed the following results:

### miRNAs differentially expressed in B-AL/B-Ly compared to NCLT

**Fig. 1:**
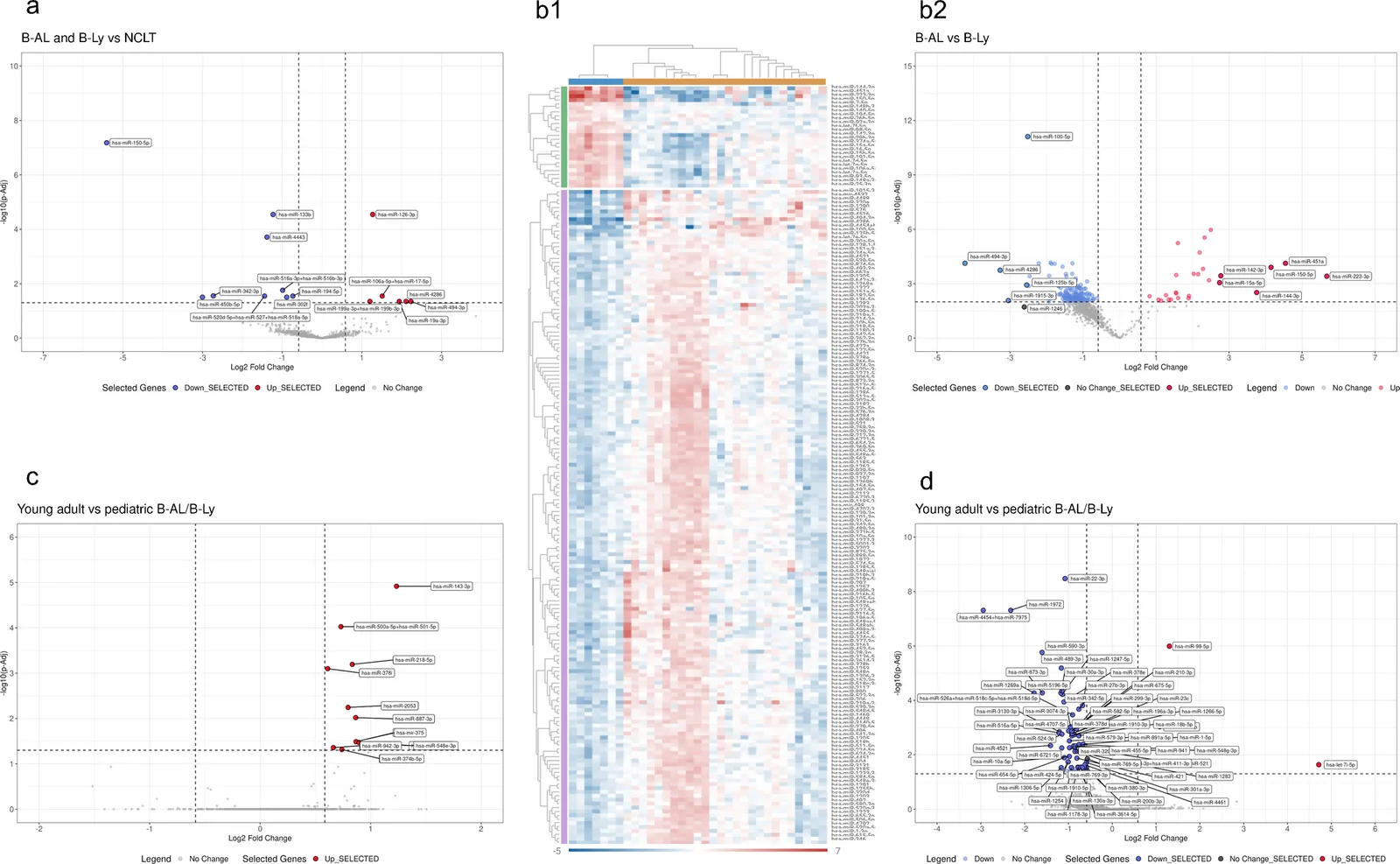
Differentially expressed miRNAs according to subgroups. Volcano plots: X-axis log_2_FC, Y-axis log_10_ *p*-adj (**a**) Volcano plot: Differential miR expression in B-AL and B-Ly versus NCLT. (**b1**) Heatmap showing differential miRNA expression in B-Ly and B-AL (FC > 1.5, *p-*adj < 0.01). Differential miRNA expression intensity: red color tones upregulation, blue color tones downregulation. X-axis: B-AL cases (blue) B-Ly (orange), Y-axis differentially expressed miRNAs (green: upregulated in B-AL, violet: downregulated in B-AL). Bottom: reference bar with log2-FC scale of expression values. (**b2**) Volcano plot: Differential miR expression in B-AL versus B-Ly. (**c**) Volcano plot: Differential miR expression in B-AL and B-Ly in young adults versus pediatric age group. (**d**) Volcano plot: Differential miR expression in adverse versus favorable outcome B-AL and B-Ly

Expression profiling analysis of B-AL and B-Ly (*n* = 33) compared to a pool of NCLT (*n* = 4) showed 20 miRNAs which were differentially expressed (DE) with fold change (FC) > 1.5, *p*-adj < 0.05. Eight miRNAs were differentially upregulated in B-AL/B-Ly compared to NCLT and 12 miRNAs were downregulated (supplementary Table 1). The higher expressed miRNAs in B-AL and B-Ly compared to NCLT were, among others, miR-494-3p, miR-4286, miR-19a-3p, miR-106a-5p, miR-17-5p and miR-126-3p. The lower expressed miRNAs in B-AL and B-Ly compared to NCLT included miR-150-5p, miR-450b-5p and miR-342-3p among others (Fig. 1a).

MiRNA expression of B-Ly (*n* = 26) was compared to NCLT (*n* = 4) and revealed 23 miRNAs which were DE with FC > 1.5 and *p*-adj < 0.05. Seven miRNAs were differentially upregulated in B-Ly compared to NCLT and 16 miRNAs were downregulated (supplementary Table 1). The higher expressed miRNAs in B-Ly compared to NCLT included, among others, miR-494-3p, miR-4286, miR-199a-3p, miR-199b-3p, miR-126-3p, and miR-378i; the lower expressed miRNAs included miR-450b-5p, miR-342-3p, miR-223-3p, and miR-150-5p. Expression profiling analysis of B-AL (*n* = 7) compared to NCLT (*n* = 4) showed 39 miRNAs which were DE with FC > 1.5, *p*-adj < 0.05. 12 miRNAs were differentially upregulated in B-AL compared to NCLT and 27 miRNAs were downregulated (supplementary Table 1). The higher expressed miRNAs in B-AL compared to NCLT included, among others, miR-144-3p, miR-19a-3p, miR-106a-5p, miR-17-5p, miR-451a, miR-20a-5p, and miR-20b-5p; the lower expressed miRNAs included miR-450b-5p, miR-155-5p, miR-135b-5p, miR-125b-5p, and miR-150-5p. A significant upregulation of miR-17-5p, miR-19a-3p, miR-19b-3p, and miR-20a-5p, part of the miR-17 ~ 92 cluster, was observed in B-AL samples compared to NCLT (FC 7.98; FC 8.78; FC 4.78; and FC 5.25, respectively, *p*-adj < 0.05) (supplementary Table 2).

### miRNAs differentially expressed in B-AL/B-Ly according to clinicopathological subgroups

Expression profiling analysis comparing B-AL (*n* = 7) with B-Ly (*n* = 26) showed that B-AL and B-Ly segregate into two major miRNA clusters (Fig. 1b1). 199 miRNAs were found to be DE in B-AL compared with B-Ly with FC > 1.5, *p-*adj < 0.01, 27 upregulated and 172 downregulated (supplementary Table 3). The higher expressed miRNAs in B-AL compared to B-Ly were, among others, miR-223-3p, miR-451a, miR-150-5p, miR-144-3p, miR-142-3p and miR-15a-5p, whereas the lower expressed miRNAs in B-AL compared to B-Ly included, among others, miR-494-3p, miR-4286, miR-1915-3p, miR-125b-5p, and miR-100-5p (Fig. 1b2).

miRNA expression profiling analysis in B-Ly by stage, CNS and bone marrow involvement as well as by EBV-status did not show statistically significant results after multiple comparison adjustment (supplementary Tables 4 and 5). When comparing the EBV-positive B-Ly (*n* = 5) and EBV-negative B-Ly subgroups (*n* = 21), three differentially expressed miRNAs not reaching statistical significance after multiple comparison adjustment were identified, with upregulation of miR-139-5p (FC 1.95, *p*-value 0.04, *p*-adj 0.99) and downregulation of miR-27b-3p (FC-1.29, *p*-value 0.03, *p*-adj 0.99) and miR-320e (FC-1.55, *p*-value 0.03, *p*-adj 0.99) (supplementary Table 5).

Exploratory analysis of miRNA profiling in B-AL and B-Ly regarding age (range 3–18 years), comparing the young adult (18 years old, *n* = 2)) versus the pediatric age group (< 18 years, *n* = 31) showed 11 miRNAs differentially upregulated with FC > 1.5, *p-*adj < 0.05. miR-143-3p, miR-548e-3p and miR-375 were, among others, the higher expressed miRNAs in patients with B-AL/B-Ly in the young adult group (Fig. 1c, supplementary Table 6).

When analyzing differential miRNA expression in B-AL/B-Ly with adverse (*n* = 2) versus favorable outcome (*n* = 31), 75 miRNAs were found to be DE with FC > 1.5, *p-*adj < 0.05, three upregulated and 72 downregulated. The higher expressed miRNAs in B-AL/B-Ly with adverse outcome were let-7i-5p, miR-98-5p and miR-7-5p, whereas the lower expressed miRNAs were, among others, miR-4454, miR-7975, miR-1972, miR-1269a, and miR-10a-5p (Fig. 1d, supplementary Table 7).

### miRNA validation by qPCR analysis

**Fig. 2:**
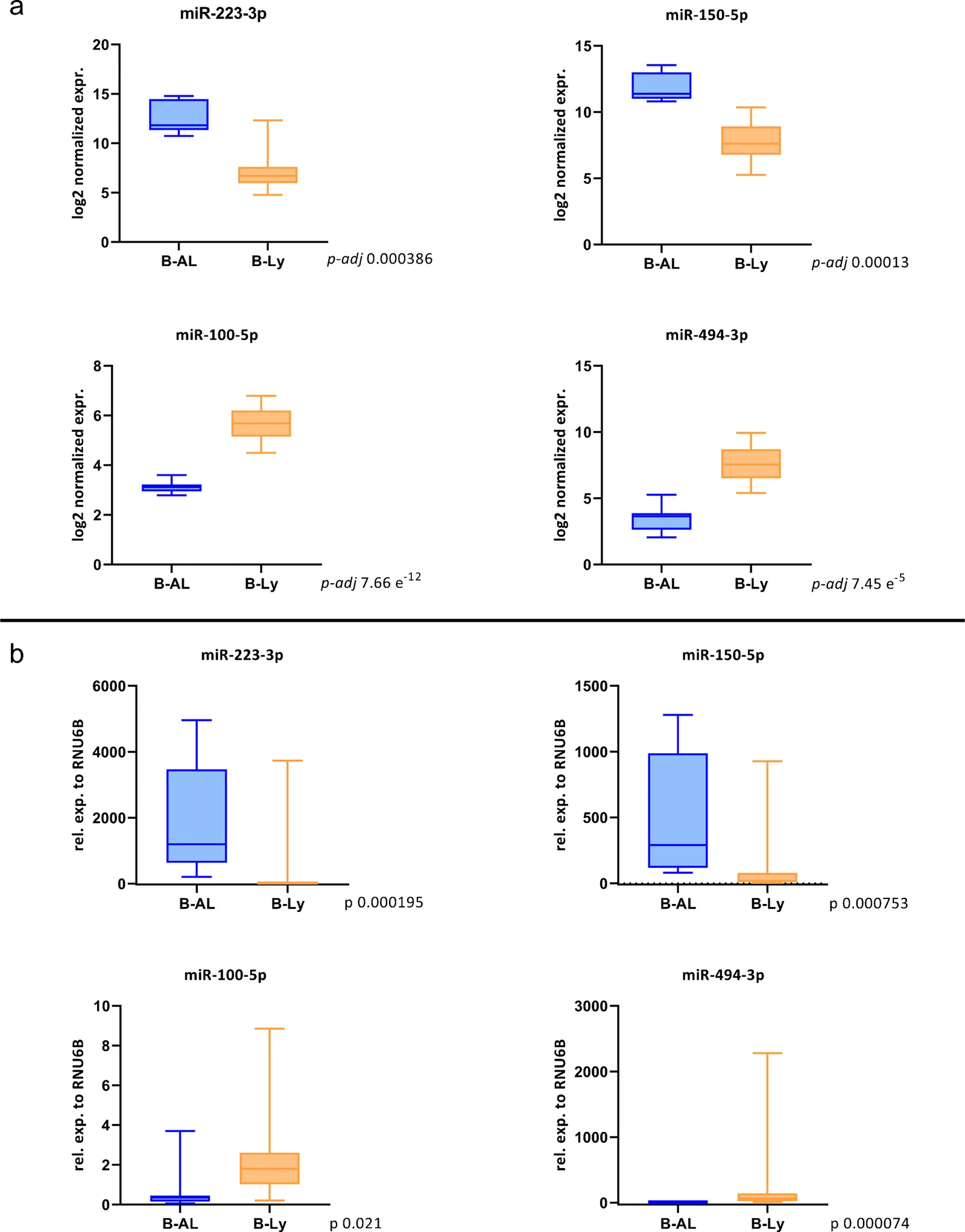
miRNA validation by qPCR analysis. (**a**) Box-plots. Log2 normalized expression of miR-223-3p, miR-150-5p, miR-100-5p and miR-494-3p in Nanostring miRNA microarrays in B-AL versus B-Ly. (**b**) qPCR analysis. miR-223-3p, miR-150-5p, miR-100-5p and miR-494-3p relative expression to RNU6B in B-AL versus B-Ly

Two significantly upregulated miRNAs in B-AL compared with B-Ly, miR-223-3p (FC 51.16 (5.68log2), *p-adj* 0.000386) and miR-150-5p (FC 17.74 (4.15log2), *p-adj* 0.00013) and two significantly downregulated miRNAs in B-AL compared with B-Ly, miR-100-5p (FC −5.71 (− 2.51log2), *p*-adj 7.66-e^12^) and miR-494-3p (FC-18.82 (− 4.23log2), *p*-*adj* 7.45-e^5^), were chosen for further validation of NanoString array results using qPCR analysis with miRNA-specific TaqMan assays against the endogenous control RNU6B. Consistency of miRNA-array screening results was validated for miR-223-3p, miR-150-5p, miR-100-5p and miR-494-3p, with a median FC in B-AL against B-Ly of 99.4, 15.6, − 5.1 and − 9.4 with *p*-values of 0.000195, 0.000753, 0.021 and 0.000074 respectively (Figs. 2a, b, supplementary Table 8).

### Bioinformatic analysis of validated target genes and pathways

**Fig. 3:**
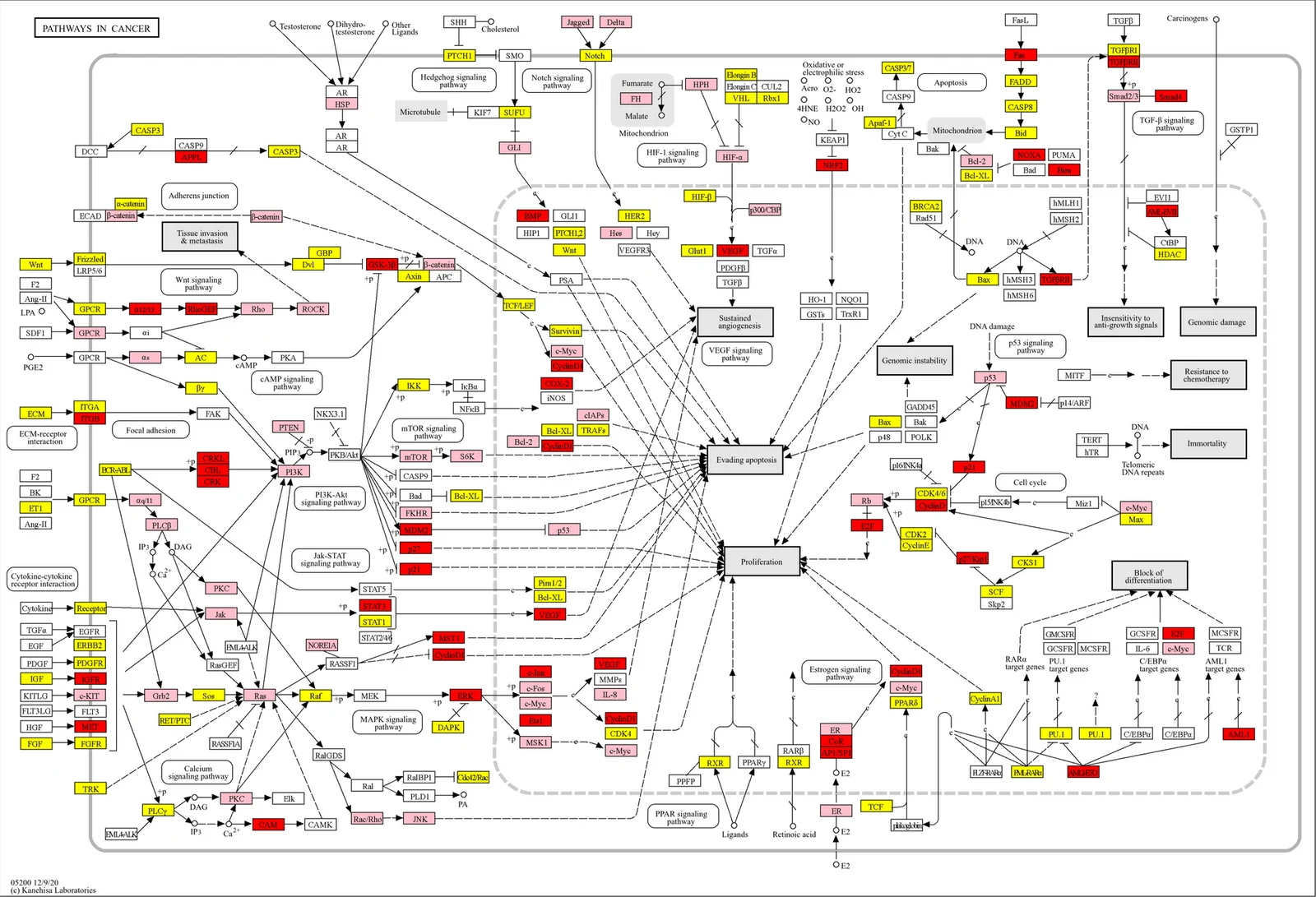
In silico predicted target genes and involved KEGG cancer signaling pathways of DE miRNAs in B-AL/B-Ly compared to NCLT (*p*-value 1.80 e^−13^, FDR 2.04 e.^−11^; DIANA-miRPath v4 platform-miRbase v22.1). Targets are highlighted according to the number of targeting miRNAs (yellow:1, pink:2, red: >  = 3) on KEGG map “pathways in cancer”

DE miRNAs in B-AL and B-Ly compared to NCLT (FC > 1.5, *p*-adj < 0.05; supplementary Table 1) were evaluated in silico regarding their experimentally validated target transcripts and involved signaling pathways through bioinformatic analysis with the Rosalind® and DIANA-miRPath v4® KEGG pathway platforms. The number of validated targeted transcripts by up- and downregulated miRNAs of the B-AL and B-Ly versus NCLT miRNA signature reaches 10,824 (supplementary file, Table 9). According to the DIANA-miRPath v4®-miRbase v22.1 platform, the main predicted signaling pathways in cancer-related KEGG pathways targeted by these miRNAs are the MAPK, PI3K-Akt, JAK-STAT, VEGF, TP53, Fas, TGF-β and MYC signaling pathways (*p*-value 1.80 e-^13^, FDR 2.04 e-^11^) (Fig. 3).

## Discussion

A translational gap exists in B-AL and B-Ly regarding miRNAs associated with clinicopathological features and outcome. In the present study new miRNA signatures of relevance in B-AL and B-Ly could be recognized. Analysis of miRNAs deregulated in B-AL/B-Ly compared to NCLT identified a consistent set of twenty differentially expressed miRNAs (FC > 1.5, *p*-adj < 0.05). In silico, the main predicted signaling pathways in cancer-related KEGG pathways targeted by this set of miRNAs include the MAPK, PI3K-Akt, JAK-STAT, VEGF, TP53, Fas, TGF-β, and MYC signaling pathway (*p*-value 1.80 e-^13^, FDR 2.04 e-^11^), which are known to be deregulated pathways in B-Ly [11], illustrating a potential biological impact of these miRNAs in the pathogenesis of the disease at the posttranscriptional regulatory level. Recent data suggest that EBV-positive B-Ly and EBV-negative B-Ly form separate biologic subgroups [15–17]. EBV-encoded miRNAs exert oncogenic effects by modulating cancer signaling pathways as well as the host immune response, thereby facilitating viral persistence and tumorogenesis [33–41]. Virus-encoded miRNAs contribute to the molecular profile of EBV-positive B-Ly [42]. In this cohort, the comparison of EBV-positive and EBV-negative B-Ly yielded no DE host miRNAs with statistical significance after multiple comparison adjustment. Small cohort size, a low number of EBV-positive B-Ly cases and the fact that viral EBV-miRNAs are not represented in the Nanostring v3 miRNA array might contribute to the lack of statistically significant differences observed in the comparison of EBV-positive and EBV-negative B-Ly in this cohort.

In this study, comparison of B-Ly to NCLT revealed among the differentially expressed miRNAs upregulation of miR-494-3p as well as significant downregulation of miR-223-3p and miR-150-5p (FC > 1.5, *p-adj* < 0.05). Differential upregulation of miR-494-3p as well as downregulation of miR-150-5p and miR-223-3p has been reported in B-Ly with regard to other MYC-driven lymphomas [25], reflecting a possible role of these miRNAs both in the physiopathology and as potential biomarkers of B-Ly with regard to other NHL. miR-494-3p is part of a negative regulatory loop of *MYC* modulated by H3K27me3 and acts as a repressor of *MYC* expression in B-Ly cells [43]. miR-150-5p contributes to terminal B-cell differentiation in vitro [44] and modulates among others c-MYB [44], Survivin [45], FOXP1 [45], FLT3 [46], BCR-signaling [47] and ZDHHC11 [27, 48].

miRNA expression profiling in B-AL compared to NCLT showed significant upregulation of components of the miR-17∼92 cluster (FC > 1.5, *p-*adj < 0.05). Significant upregulation of miR-17a-5p and miR-19a-3p as well as non-significant change in miR-18a-5p expression are concordant with published results on Ago-2 IP in B-Ly [49]. Upregulation of a truncated version of miR-17∼92 has been described not only in B-Ly [50, 51], but also in other B-cell lymphomas, including diffuse large B-cell lymphoma, mantle cell lymphoma and chronic lymphocytic leukemia [52, 53]. In B-Ly cell lines, miR-17∼92 expression in combination with *MYC* expression is characterized by a repression of apoptosis that was otherwise present in *MYC*-induced lymphomas [53]. Recent studies have shown that *TP53* mutations have high prognostic value in B-Ly, particularly in conjunction with gains of GPC5/MIR17HG [11].

A consistent miRNA expression pattern was recognized when comparing B-AL and B-Ly, showing that B-AL and B-Ly segregate into two major miRNA clusters, with a set of 199 differentially expressed miRNAs (FC > 1.5, *p*-adj < 0.01). To the best of our knowledge, differentially expressed miRNA signatures in B-AL and B-Ly have not been described so far. Differences between B-AL and B-Ly have also been reported at the genomic [11] and transcriptomic [8] level, showing that *CCND3* mutations are detected more frequently in B-AL, whereas *GNAI2*, *RFX7*, *P2RY8*, *DDX3X* and *GNA13* aberrations are predominantly found in the B-Ly [11]. At the transcriptomic level [8], B-AL displays an expression pattern more typical for non-molecular B-Ly for the majority of genes in the molecular B-Ly classifier developed to classify B-Ly in solid tumor presentation. The potential biological role of these miRNAs on pathway regulation and tumor microenvironment modulation at the posttranscriptional level contributing to the leukemic and lymphomatous presentation needs to be further elucidated experimentally in follow-up studies.

When comparing the results of this study with recently published data on miRNA sequencing in pediatric B-Ly, significant upregulation of miR-126-3p, miR-199a-3p and miR-199b-3p, as well as downregulation of miR-4443 was reported in B-Ly tumor tissue compared to centroblasts of obstructive sleep apnea tonsils [54, 55], which is in line with our results comparing B-AL/B-Ly to NCLT, showing significant upregulation of miR-126-3p, miR-199a-3p and miR-199b-3p along with downregulation of miR-4443, among others (FC > 1.5, *p*-adj < 0.05).

In this CAYA-cohort of B-AL and B-Ly a set of 11 differentially expressed miRNAs was observed in an exploratory analysis between age groups older and younger than 18 years of age (FC > 1.5, *p-*adj < 0.05). Molecular age-related differences in B-Ly have been extensively described, with a higher frequency of mutations in *ID3*, *DDX3X*, *ARID1A* and *SMARCA4* in pediatric patients, while *BCL2* and *YY1AP1* are almost exclusively mutated in adult patients [11].

Analysis of miRNA signatures by outcome, although limited by the small patient number and the inclusion of one de novo B-AL and one relapsed B-Ly case with unfavorable outcome, revealed a set of DE miRNAs between cases with adverse and favorable outcome. Notably, significant downregulation of miR-10a-5p was observed (FC − 1.97, *p-*adj 0.01) in the unfavorable outcome group. Although this observation is limited by the small sample size, significant downregulation of miR-10a-5p associated with an adverse outcome in B-Ly has previously been reported [56] and confers validity to these findings. As evaluated by Oduor et al., high expression of CD59 as a result of miR-10a-5p imbalance in B-Ly may determine reduced sensitivity to chemo- and immunotherapy and explain treatment failure and reduced overall survival [56]. Further studies determining the impact of miRNA signatures in relapsed B-AL/B-Ly are required.

Despite the utility of in silico target prediction tools, the posttranscriptional regulatory impact of miRNAs needs to be validated experimentally. miRNA-target interactions underlie complex dynamic mechanisms, including mutual stochiometric effects between miRNA and mRNA targets, differences in efficiency of miRNA–target interactions, targeting of one mRNA by multiple, both by up- and downregulated miRNAs as well as dissimilarities in cell type specific miRNA interaction partners [21]. Therefore, experimental validation of miRNA-target interactions in B-AL and B-Ly is warranted in further studies.

Although comparable to other studies on miRNA signatures in different entities, a limitation of this study is the small patient number, limiting the robustness of the results of the study. Evaluation through miRNA sequencing may provide a better insight into some further undetected significantly expressed miRNAs not covered by the assay used.

Nevertheless, in the present study a comprehensive analysis of miRNA profiles by miRNA arrays which was further validated by qPCR allowed us to identify miRNA signatures of relevance in B-AL and B-Ly. The results of this exploratory kick-off study shall pave the way for further studies evaluating miRNAs as diagnostic and prognostic markers in B-AL and B-Ly in larger cohorts. Next steps include clinical validation, ideally by RNA-sequencing in independent cohorts, as well as functional validation of differentially expressed miRNAs in cell line models including gain- or loss-of-function studies.

In summary, miRNAs deregulated in B-AL/B-Ly compared to NCLT as well as miRNA signatures related to leukemic presentation in B-AL/B-Ly could be recognized in this study. Prospective studies in larger cohorts are required to further validate these findings.

## Supplementary Information

Below is the link to the electronic supplementary material.

ESM 1 (DOCX 66.2 KB)

ESM 2 (XLSX 302 KB)

## Data Availability

Data are provided within the manuscript and supplementary information files. The datasets generated and analyzed during the current study are available in the NCBI’s Gene Expression Omnibus, accession number GSE296370, (https://www.ncbi.nlm.nih.gov/geo/query/acc.cgi?acc=GSE296370) upon acceptance and through the corresponding author on request.
