# Supplementary material for: miRNA profiling in pediatric and young adult Burkitt leukemia and lymphoma
Source: Virchows Arch. 2026 Feb 6;489(4):941–51. doi: 10.1007/s00428-026-04426-0 (PMC13714106; doi:10.1007/s00428-026-04426-0)
Supplement: Supplementary file 1 [file 428_2026_4426_MOESM1_ESM.docx]

Supplementary table and file legend:

**Supplementary table 1** miRNAs differentially expressed in B-Ly and B-AL versus NCLT (FC>1.5, *p*-adj<0.05)

**Supplementary table 2** miR-17∼92 cluster in B-AL and B-Ly compared to NCLT

**Supplementary table 3** Differentially expressed miRNAs in B-AL (n=7) and B-Ly (n=26) (FC>1.5, *p*-adj<0.01)

**Supplementary table 4** Differentially expressed miRNAs in B-AL (n=7) and B-Ly (n=26) according to stage and clinical characteristics (FC>1.5, *p*<0.05)

**Supplementary table 5 Differentially expressed miRNAs in EBV(+) (n=5) and EBV(-) (n=21) B-Ly (FC>1.5, *p*<0.05).**

**Supplementary table 6** Differentially expressed miRNAs in B-AL and B-Ly regarding adult (>=18 years old) (n=2) versus pediatric (<18 years) age (n=31) (FC>1.5, *p*-adj<0.05)

**Supplementary table 7** Differentially expressed miRNAs in B-AL and B-Ly with adverse outcome (n=2) versus favorable outcome (n=31) (FC>1.5, *p*-adj<0.05)

**Supplementary table 8** qPCR analysis for miR-223-3p, miR-150-5p, miR-100-5p and miR-494-3p against the endogenous control RNU6B

**Supplementary file table 9** Validated target transcripts of differentially expressed miRNAs in B-AL and B-Ly compared to NCLT (Excel file)

**Supplementary file 1** B-AL, B-Ly, NCLT data sheet

**Supplementary table 1** **miRNAs differentially expressed in B-Ly and in B-AL versus NCLT (FC>1.5, p-adj<0.05)**

| **B-AL/ B-LY (N=33) versus NCLT (N=4)** |  |  |  |
| --- | --- | --- | --- |
| **Name** | **FC** | **p-value** | **p-adj** |
| miR-494-3p | 4.70039 | 0.00084 | 0.04482 |
| miR-4286 | 4.31416 | 0.00083 | 0.04482 |
| miR-19a-3p | 3.83206 | 0.00080 | 0.04482 |
| miR-106a-5p+miR-17-5p | 2.84909 | 0.00032 | 0.02841 |
| miR-126-3p | 2.41381 | 1.07e-7 | 2.85e-5 |
| miR-199a-3p+miR-199b-3p | 2.30212 | 0.00069 | 0.04482 |
| miR-194-5p | -1.66223 | 0.00032 | 0.02841 |
| miR-302f | -1.84992 | 0.00042 | 0.03125 |
| miR-516a-3p+miR-516b-3p | -1.9847 | 0.00011 | 0.01730 |
| miR-133b | -2.34383 | 8.41e-8 | 2.85e-5 |
| miR-4443 | -2.60805 | 9.72e-7 | 0.00019 |
| miR-520d-5p+miR-527+miR-518a-5p | -2.72076 | 0.00026 | 0.02841 |
| miR-342-3p | -6.63814 | 0.00021 | 0.02783 |
| miR-450b-5p | -8.01626 | 0.00043 | 0.03125 |
| miR-150-5p | -42.4552 | 8.24e-11 | 6.57e-8 |
| **B-LY (N=26) versus NCLT (N=4)** |  |  |  |
| **Name** | **FC** | **p-value** | **p-adj** |
| miR-494-3p | 8.5165 | 0.00012 | 0.01343 |
| miR-4286 | 6.78505 | 0.00022 | 0.01584 |
| miR-199a-3p+miR-199b-3p | 2.7533 | 0.00016 | 0.01401 |
| miR-126-3p | 2.64761 | 3.92e-8 | 1.56e-5 |
| miR-378i | 1.88536 | 0.00055 | 0.03139 |
| miR-378e | 1.6668 | 0.00089 | 0.03952 |
| miR-140-5p | -1.66418 | 5.84e-6 | 0.00093 |
| miR-516a-3p+miR-516b-3p | -1.85571 | 0.00033 | 0.02046 |
| miR-194-5p | -1.92969 | 0.00024 | 0.01584 |
| miR-133b | -2.14322 | 1.99e-6 | 0.00053 |
| miR-506-3p | -2.38905 | 0.00104 | 0.04350 |
| miR-450a-5p | -2.45978 | 0.00071 | 0.03327 |
| miR-520d-5p+miR-527+miR-518a-5p | -2.66915 | 0.00014 | 0.01343 |
| miR-4443 | -2.8186 | 3.54e-6 | 0.00071 |
| miR-451a | -3.93682 | 0.00067 | 0.03327 |
| miR-450b-5p | -7.24211 | 0.00022 | 0.01584 |
| miR-342-3p | -8.35251 | 0.00064 | 0.03327 |
| miR-223-3p | -11.1453 | 9.52e-5 | 0.01267 |
| miR-150-5p | -80.3825 | 6.99e-9 | 5.58e-6 |
| **B-AL (N=7) versus NCLT (N=4)** |  |  |  |
| **Name** | **FC** | **p-value** | **p-adj** |
| miR-144-3p | 24.1157 | 0.00014 | 0.01636 |
| miR-19a-3p | 8.77951 | 0.00014 | 0.01636 |
| miR-106a-5p+miR-17-5p | 7.98831 | 6.33e-5 | 0.01636 |
| miR-451a | 6.08995 | 0.00075 | 0.03505 |
| miR-20a-5p+miR-20b-5p | 5.25048 | 0.00100 | 0.03831 |
| miR-19b-3p | 4.7789 | 0.00195 | 0.04867 |
| miR-7-5p | 4.32479 | 8.93e-6 | 0.00713 |
| miR-148a-3p | 4.04713 | 0.00025 | 0.01809 |
| miR-374a-5p | 3.63511 | 0.00106 | 0.03831 |
| miR-106b-5p | 2.68637 | 0.00124 | 0.04313 |
| miR-4443 | -2.17348 | 0.00011 | 0.01636 |
| miR-30b-5p | -2.17662 | 0.00192 | 0.04867 |
| miR-203a-3p | -2.19967 | 0.00210 | 0.04925 |
| miR-105-5p | -2.24694 | 0.00104 | 0.03831 |
| miR-1285-5p | -2.50689 | 0.00015 | 0.01636 |
| miR-136-5p | -2.6044 | 0.00154 | 0.04867 |
| miR-574-5p | -2.60978 | 0.00055 | 0.02920 |
| miR-642a-3p | -2.72095 | 0.00084 | 0.03706 |
| miR-516a-3p+miR-516b-3p | -2.83261 | 9.57e-5 | 0.01636 |
| miR-3202 | -3.04305 | 0.00100 | 0.03831 |
| miR-520d-5p+miR-527+miR-518a-5p | -3.24845 | 0.00020 | 0.01636 |
| miR-138-5p | -3.25857 | 0.00166 | 0.04867 |
| miR-133b | -3.63373 | 0.00020 | 0.01636 |
| miR-488-3p | -3.72477 | 0.00135 | 0.04492 |
| miR-497-5p | -3.8476 | 0.00171 | 0.04867 |
| miR-520g-3p | -3.88367 | 0.00177 | 0.04867 |
| miR-378c | -3.91795 | 0.00046 | 0.02599 |
| miR-206 | -4.11291 | 0.00202 | 0.04887 |
| miR-219a-5p | -4.15757 | 0.00035 | 0.02154 |
| miR-150-5p | -4.40863 | 0.00163 | 0.04867 |
| miR-125b-5p | -4.67897 | 0.00035 | 0.02154 |
| miR-135b-5p | -4.99401 | 0.00186 | 0.04867 |
| miR-155-5p | -11.235 | 0.00073 | 0.03505 |
| miR-450b-5p | -12.9983 | 0.00017 | 0.01636 |

**Supplementary table 2** **miR-17∼92 cluster in B-AL and B-Ly compared to NCLT**

| **miR-17∼92 cluster** |  |  |  |  |  |  |  |
| --- | --- | --- | --- | --- | --- | --- | --- |
| B-AL (N=7) | FC | *p-value* | *p-adj* | B-LY (N=26) | FC | *p-value* | *p-adj* |
| miR-17-5p | 7.98831 | 6.33e-5 | 0.01636 |  | 2.09773 | 0.00813 | 0.20022 |
| miR-18a-5p | 1.46159 | 0.27366 | 0.38651 |  | 1.34902 | 0.29159 | 0.77050 |
| miR-19a-3p | 8.77951 | 0.00014 | 0.01636 |  | 2.97915 | 0.00342 | 0.10153 |
| miR-19b-3p | 4.7789 | 0.00195 | 0.04867 |  | 1.89018 | 0.08990 | 0.57391 |
| miR-20a-5p | 5.25048 | 0.00100 | 0.03831 |  | 1.84867 | 0.06969 | 0.53291 |
| miR-92a-1-5p | -1.31979 | 0.32144 | 0.42752 |  | -1.1338 | 0.48874 | 0.84055 |

**Supplementary table 3 Differentially expressed miRNAs in B-AL (n=7) and B-Ly (n=26) (FC>1.5, p-adj<0.01)**

| **Name** | **FC** | ***p*-value** | ***p*-adj** |
| --- | --- | --- | --- |
| miR-223-3p | 51.1567 | 1.23e-5 | 0.00039 |
| miR-451a | 23.3309 | 8.43e-7 | 7.45e-5 |
| miR-150-5p | 17.743 | 2.19e-6 | 0.00013 |
| miR-144-3p | 13.5061 | 0.00025 | 0.00306 |
| miR-142-3p | 6.84177 | 1.05e-5 | 0.00036 |
| miR-15a-5p | 6.67987 | 4.24e-5 | 0.00087 |
| miR-16-5p | 5.65325 | 2.70e-9 | 1.08e-6 |
| miR-29b-3p | 5.46386 | 2.49e-5 | 0.00065 |
| miR-15b-5p | 5.02325 | 1.08e-8 | 2.86e-6 |
| let-7g-5p | 4.87712 | 2.86e-6 | 0.00015 |
| let-7a-5p | 4.56951 | 1.19e-7 | 1.90e-5 |
| miR-191-5p | 4.44604 | 7.41e-6 | 0.00030 |
| miR-25-3p | 4.08818 | 6.14e-6 | 0.00028 |
| miR-374a-5p | 3.73744 | 0.00060 | 0.00471 |
| miR-106a-5p+miR-17-5p | 3.70574 | 0.00100 | 0.00628 |
| miR-93-5p | 3.03717 | 0.00095 | 0.00615 |
| let-7d-5p | 3.02581 | 2.88e-8 | 5.74e-6 |
| let-7f-5p | 2.9338 | 0.00032 | 0.00343 |
| miR-7-5p | 2.9316 | 8.78e-6 | 0.00033 |
| miR-148a-3p | 2.91273 | 0.00025 | 0.00305 |
| miR-98-5p | 2.65621 | 0.00152 | 0.00755 |
| miR-92a-3p | 2.54134 | 0.00140 | 0.00743 |
| miR-26b-5p | 2.41382 | 0.00049 | 0.00427 |
| miR-140-5p | 2.20523 | 0.00190 | 0.00866 |
| miR-148b-3p | 2.09468 | 0.00150 | 0.00750 |
| miR-194-5p | 1.7683 | 0.00063 | 0.00487 |
| miR-1180-3p | -1.53233 | 0.00100 | 0.00628 |
| miR-27b-3p | -1.64517 | 0.00149 | 0.00748 |
| miR-101-3p | -1.64552 | 0.00115 | 0.00668 |
| miR-542-5p | -1.68089 | 0.00053 | 0.00437 |
| miR-548d-5p | -1.68326 | 0.00193 | 0.00870 |
| miR-362-3p | -1.76577 | 0.00035 | 0.00364 |
| miR-3140-5p | -1.77055 | 0.00155 | 0.00758 |
| miR-342-5p | -1.7816 | 0.00052 | 0.00434 |
| miR-320d | -1.83405 | 0.00165 | 0.00793 |
| miR-4707-3p | -1.83909 | 0.00104 | 0.00642 |
| miR-182-5p | -1.84745 | 0.00028 | 0.00330 |
| miR-151a-3p | -1.85394 | 3.28e-5 | 0.00075 |
| miR-1283 | -1.86393 | 0.00176 | 0.00823 |
| miR-496 | -1.87895 | 0.00039 | 0.00381 |
| miR-31-5p | -1.91054 | 0.00013 | 0.00188 |
| miR-580-3p | -1.91817 | 0.00212 | 0.00923 |
| miR-3131 | -1.95077 | 0.00120 | 0.00679 |
| miR-758-3p+miR-411-3p | -1.9614 | 0.00042 | 0.00392 |
| miR-152-3p | -1.96218 | 0.00014 | 0.00191 |
| miR-4421 | -1.98843 | 0.00123 | 0.00679 |
| miR-122-5p | -1.99405 | 0.00220 | 0.00934 |
| miR-30a-5p | -2.00505 | 0.00088 | 0.00597 |
| miR-3065-5p | -2.01601 | 0.00031 | 0.00339 |
| miR-627-5p | -2.01787 | 0.00221 | 0.00934 |
| miR-584-5p | -2.03547 | 0.00120 | 0.00679 |
| miR-379-5p | -2.0525 | 0.00167 | 0.00796 |
| miR-615-5p | -2.06287 | 0.00076 | 0.00549 |
| miR-139-3p | -2.06888 | 0.00031 | 0.00339 |
| miR-378h | -2.07346 | 0.00169 | 0.00804 |
| miR-1469 | -2.07939 | 0.00014 | 0.00191 |
| miR-3182 | -2.08005 | 0.00142 | 0.00748 |
| miR-455-3p | -2.08168 | 0.00020 | 0.00262 |
| miR-489-3p | -2.08947 | 5.80e-5 | 0.00116 |
| miR-422a | -2.09483 | 0.00083 | 0.00579 |
| miR-655-3p | -2.10053 | 0.00039 | 0.00382 |
| miR-105-5p | -2.10323 | 0.00110 | 0.00654 |
| miR-539-3p | -2.11666 | 0.00060 | 0.00471 |
| miR-136-5p | -2.12249 | 0.00010 | 0.00169 |
| miR-1257 | -2.12533 | 0.00210 | 0.00922 |
| miR-1323 | -2.1324 | 0.00149 | 0.00748 |
| miR-548z+miR-548h-3p | -2.15078 | 0.00155 | 0.00758 |
| miR-518b | -2.15944 | 0.00160 | 0.00777 |
| miR-196a-5p | -2.16061 | 0.00011 | 0.00174 |
| miR-4792 | -2.16112 | 0.00175 | 0.00823 |
| miR-520a-5p | -2.17159 | 0.00112 | 0.00660 |
| miR-513c-5p | -2.17174 | 0.00049 | 0.00429 |
| miR-28-3p | -2.1752 | 0.00163 | 0.00788 |
| miR-302a-5p | -2.17851 | 0.00128 | 0.00694 |
| miR-2116-5p | -2.18034 | 0.00083 | 0.00579 |
| miR-10a-5p | -2.18094 | 0.00081 | 0.00579 |
| miR-1908-3p | -2.18545 | 0.00111 | 0.00654 |
| miR-216b-5p | -2.18806 | 0.00221 | 0.00934 |
| miR-1271-5p | -2.19967 | 0.00099 | 0.00628 |
| miR-4451 | -2.20903 | 8.90e-5 | 0.00151 |
| miR-604 | -2.21184 | 7.00e-5 | 0.00133 |
| miR-154-5p | -2.21722 | 0.00073 | 0.00544 |
| miR-548e-3p | -2.22545 | 0.00076 | 0.00549 |
| miR-1203 | -2.23642 | 0.00012 | 0.00181 |
| miR-3202 | -2.23705 | 0.00041 | 0.00387 |
| miR-548e-5p | -2.23752 | 0.00185 | 0.00850 |
| miR-574-5p | -2.24008 | 0.00123 | 0.00679 |
| miR-548q | -2.24653 | 0.00043 | 0.00392 |
| miR-339-3p | -2.2467 | 0.00221 | 0.00934 |
| miR-1262 | -2.25035 | 0.00147 | 0.00748 |
| miR-371b-5p | -2.2535 | 0.00148 | 0.00748 |
| miR-492 | -2.25633 | 0.00197 | 0.00879 |
| miR-151a-5p | -2.26423 | 0.00022 | 0.00283 |
| miR-377-3p | -2.27739 | 0.00237 | 0.00976 |
| miR-1-3p | -2.29139 | 2.75e-5 | 0.00067 |
| miR-203a-3p | -2.29315 | 1.74e-5 | 0.00051 |
| miR-1255b-5p | -2.29377 | 0.00176 | 0.00823 |
| miR-33b-5p | -2.29797 | 0.00139 | 0.00743 |
| miR-520c-3p | -2.30662 | 0.00090 | 0.00606 |
| miR-1269b | -2.3135 | 0.00041 | 0.00387 |
| miR-219a-2-3p | -2.3144 | 0.00077 | 0.00551 |
| miR-513a-5p | -2.31609 | 0.00216 | 0.00934 |
| miR-499b-3p | -2.31744 | 0.00045 | 0.00403 |
| miR-5001-3p | -2.33079 | 0.00222 | 0.00934 |
| miR-1205 | -2.33229 | 0.00022 | 0.00282 |
| miR-563 | -2.33276 | 0.00094 | 0.00615 |
| miR-521 | -2.34408 | 0.00192 | 0.00870 |
| miR-518c-3p | -2.35055 | 9.93e-5 | 0.00165 |
| miR-3185 | -2.36184 | 0.00090 | 0.00606 |
| miR-874-5p | -2.36195 | 0.00027 | 0.00324 |
| miR-1233-3p | -2.36205 | 0.00135 | 0.00730 |
| miR-369-5p | -2.36669 | 0.00105 | 0.00642 |
| miR-212-3p | -2.39265 | 3.97e-5 | 0.00087 |
| miR-1286 | -2.40771 | 0.00106 | 0.00648 |
| miR-520g-3p | -2.42063 | 0.00145 | 0.00748 |
| miR-522-3p | -2.42896 | 0.00123 | 0.00679 |
| miR-511-5p | -2.43702 | 0.00146 | 0.00748 |
| miR-1306-3p | -2.44594 | 0.00117 | 0.00671 |
| miR-4448 | -2.44777 | 8.97e-7 | 7.45e-5 |
| miR-506-5p | -2.45163 | 0.00068 | 0.00508 |
| mir-498 | -2.45629 | 0.00146 | 0.00748 |
| miR-654-3p | -2.45896 | 6.43e-6 | 0.00028 |
| miR-890 | -2.46936 | 4.07e-5 | 0.00087 |
| miR-218-5p | -2.4778 | 4.27e-5 | 0.00087 |
| miR-378e | -2.48466 | 0.00050 | 0.00431 |
| miR-888-5p | -2.49355 | 0.00143 | 0.00748 |
| miR-576-3p | -2.49471 | 0.00015 | 0.00207 |
| miR-937-3p | -2.51222 | 0.00209 | 0.00922 |
| miR-128-1-5p | -2.52461 | 0.00017 | 0.00232 |
| miR-214-3p | -2.54488 | 0.00096 | 0.00619 |
| miR-10b-5p | -2.54866 | 0.00067 | 0.00508 |
| miR-1268a | -2.55025 | 0.00215 | 0.00932 |
| miR-199a-5p | -2.56813 | 0.00128 | 0.00694 |
| miR-874-3p | -2.57022 | 0.00012 | 0.00175 |
| miR-1197 | -2.58401 | 0.00230 | 0.00950 |
| miR-3136-5p | -2.58851 | 0.00109 | 0.00654 |
| miR-624-3p | -2.59149 | 0.00196 | 0.00877 |
| miR-2113 | -2.60573 | 0.00059 | 0.00471 |
| miR-1276 | -2.61868 | 2.89e-5 | 0.00068 |
| miR-224-5p | -2.64183 | 0.00101 | 0.00628 |
| miR-1277-3p | -2.64427 | 0.00036 | 0.00375 |
| miR-4284 | -2.6639 | 0.00052 | 0.00434 |
| miR-541-3p | -2.66702 | 0.00210 | 0.00922 |
| let-7e-5p | -2.66924 | 0.00111 | 0.00654 |
| miR-219a-5p | -2.67365 | 0.00012 | 0.00175 |
| miR-939-5p | -2.70096 | 0.00063 | 0.00487 |
| miR-6720-3p | -2.71065 | 0.00030 | 0.00333 |
| miR-219a-1-3p | -2.71782 | 0.00043 | 0.00392 |
| miR-374c-5p | -2.71799 | 0.00178 | 0.00824 |
| miR-766-5p | -2.72135 | 0.00086 | 0.00594 |
| miR-6721-5p | -2.74297 | 1.26e-5 | 0.00039 |
| miR-216a-5p | -2.76303 | 0.00018 | 0.00244 |
| miR-873-3p | -2.77279 | 7.02e-6 | 0.00030 |
| miR-642a-3p | -2.78663 | 0.00029 | 0.00330 |
| miR-297 | -2.79642 | 0.00023 | 0.00283 |
| miR-1185-5p | -2.79669 | 0.00094 | 0.00615 |
| miR-1185-2-3p | -2.80506 | 0.00085 | 0.00590 |
| miR-493-3p | -2.84183 | 7.54e-5 | 0.00134 |
| miR-1291 | -2.84972 | 0.00228 | 0.00949 |
| miR-452-5p | -2.85057 | 0.00058 | 0.00471 |
| miR-3614-3p | -2.86054 | 0.00059 | 0.00471 |
| miR-1305 | -2.86243 | 0.00076 | 0.00549 |
| miR-2117 | -2.89156 | 2.50e-5 | 0.00065 |
| miR-1322 | -2.93162 | 2.52e-5 | 0.00065 |
| miR-4521 | -2.93223 | 0.00122 | 0.00679 |
| miR-346 | -2.95199 | 1.87e-6 | 0.00012 |
| miR-663a | -2.98206 | 0.00052 | 0.00434 |
| miR-219b-3p | -3.00416 | 2.60e-5 | 0.00065 |
| miR-548ai+miR-570-5p | -3.05002 | 0.00011 | 0.00174 |
| miR-4516 | -3.05108 | 0.00032 | 0.00343 |
| miR-34a-5p | -3.08665 | 0.00038 | 0.00381 |
| miR-497-5p | -3.16764 | 8.05e-5 | 0.00140 |
| miR-499a-3p | -3.17006 | 0.00043 | 0.00392 |
| miR-875-3p | -3.17017 | 7.58e-5 | 0.00134 |
| miR-548ah-5p | -3.40761 | 2.57e-5 | 0.00065 |
| miR-1290 | -3.41856 | 0.00046 | 0.00411 |
| miR-3161 | -3.42881 | 0.00091 | 0.00606 |
| miR-539-5p | -3.43012 | 1.09e-6 | 7.91e-5 |
| miR-1253 | -3.52998 | 0.00038 | 0.00381 |
| miR-1285-5p | -3.64026 | 1.21e-6 | 8.02e-5 |
| miR-4455 | -3.70985 | 5.17e-7 | 6.88e-5 |
| mir-4532 | -3.75486 | 0.00115 | 0.00668 |
| miR-1972 | -3.80759 | 1.01e-5 | 0.00036 |
| miR-4454+miR-7975 | -3.83025 | 0.00239 | 0.00978 |
| miR-206 | -3.99872 | 0.00037 | 0.00378 |
| miR-320e | -4.35671 | 0.00029 | 0.00333 |
| miR-548ar-5p | -4.64478 | 9.33e-7 | 7.45e-5 |
| miR-4488 | -5.51656 | 1.26e-5 | 0.00039 |
| miR-575 | -5.57623 | 7.35e-5 | 0.00134 |
| miR-100-5p | -5.71249 | 9.60e-15 | 7.66e-12 |
| miR-125b-5p | -5.83333 | 6.08e-5 | 0.00118 |
| miR-1915-3p | -8.26308 | 0.00185 | 0.00850 |
| miR-4286 | -9.66098 | 3.60e-6 | 0.00018 |
| miR-494-3p | -18.828 | 9.18e-7 | 7.45e-5 |

**Supplementary table 4 Differentially expressed miRNAs in B-AL (n=7) and B-Ly (n=26) according to stage and clinical characteristics (FC>1.5, p<0.05)**

| **B-Ly CNS (+) versus CNS (-)** |  |  |  |
| --- | --- | --- | --- |
| **Name** | FC | *p*-value | *p-adj* |
| miR-1260a | 2.03273 | 0.01484 | 0.99927 |
| miR-3195 | 1.80022 | 0.01776 | 0.99927 |
| miR-18a-5p | 1.74496 | 0.02484 | 0.99927 |
| miR-539-5p | 1.52715 | 0.00114 | 0.91152 |
| miR-425-5p | 1.51392 | 0.01424 | 0.99927 |
| miR-150-5p | -2.52694 | 0.01936 | 0.99927 |
| **B-AL CNS (+) versus CNS (-)** |  |  |  |
| miR-577 | 2.37232 | 0.01332 | 0.99928 |
| miR-545-3p | 2.25649 | 0.03863 | 0.99928 |
| miR-638 | 2.01074 | 0.04073 | 0.99928 |
| miR-555 | 1.64421 | 0.04304 | 0.99928 |
| miR-641 | 1.55994 | 0.04838 | 0.99928 |
| miR-625-5p | 1.53651 | 0.01525 | 0.99928 |
| miR-671-3p | 1.50535 | 0.03018 | 0.99928 |
| miR-222-3p | -1.75535 | 0.04683 | 0.99928 |
| miR-193b-3p | -2.11792 | 0.02865 | 0.99928 |
| miR-150-5p | -3.63363 | 0.01345 | 0.99928 |
| **B-Ly BM<25% (+) versus BM (-)** |  |  |  |
| miR-361-5p | 1.67753 | 0.00581 | 0.99930 |
| miR-23b-3p | 1.58024 | 0.04158 | 0.99930 |
| miR-199b-5p | 1.52719 | 0.01672 | 0.99930 |
| miR-23a-3p | 1.50161 | 0.01476 | 0.99930 |
| miR-548ar-5p | -1.64643 | 0.01739 | 0.99930 |
| **B-Ly stage IV versus stages I-III** |  |  |  |
| miR-1260a | 2.03273 | 0.01484 | 0.99927 |
| miR-3195 | 1.80022 | 0.01776 | 0.99927 |
| miR-18a-5p | 1.74496 | 0.02484 | 0.99927 |
| miR-539-5p | 1.52715 | 0.00114 | 0.91152 |
| miR-425-5p | 1.51392 | 0.01424 | 0.99927 |
| miR-150-5p | -2.52694 | 0.01936 | 0.99927 |

**Supplementary table 5 Differentially expressed miRNAs in EBV(+) (n=5) and EBV(-) (n=21) B-Ly (FC>1.5, *p*<0.05)**

| **Name** | FC | *p*-value | *p-adj* |
| --- | --- | --- | --- |
| miR-139-5p | 1.95691 | 0.041 | 0.99 |
| miR-27b-3p | -1.29655 | 0.039 | 0.99 |
| miR-320e | -1-55438 | 0.038 | 0.99 |

**Supplementary table 6 Differentially expressed miRNAs in B-AL and B-Ly regarding adult (>=18 years old) (n=2) versus pediatric (<18 years) age (n=31) (FC>1.5, p-adj<0.05)**

| **Name** | **FC** | ***p-*value** | ***p-adj*** |
| --- | --- | --- | --- |
| miR-143-3p | 2.35233 | 1.52e-8 | 1.21e-5 |
| miR-548e-3p | 1.85826 | 0.00034 | 0.03383 |
| miR-375 | 1.82446 | 0.00028 | 0.03238 |
| miR-887-3p | 1.82096 | 7.18e-5 | 0.00955 |
| miR-218-5p | 1.78228 | 2.40e-6 | 0.00064 |
| miR-2053 | 1.7358 | 3.57e-5 | 0.00569 |
| miR-374b-5p | 1.66973 | 0.00061 | 0.04838 |
| miR-500a-5p+miR-501-5p | 1.65977 | 2.36e-7 | 9.43e-5 |
| miR-942-3p | 1.58307 | 0.00049 | 0.04390 |
| miR-378i | 1.52755 | 3.99e-6 | 0.00080 |

**Supplementary table 7 Differentially expressed miRNAs in B-AL and B-Ly with adverse outcome (n=2) versus favorable outcome (n=31) (FC>1.5, p-adj<0.05)**

| **Name** | **FC** | ***p*-value** | ***p-adj*** |
| --- | --- | --- | --- |
| let-7i-5p | 26.2379 | 0.00148 | 0.02313 |
| miR-98-5p | 2.46818 | 5.13e-9 | 1.02e-6 |
| miR-7-5p | 2.11741 | 1.94e-5 | 0.00093 |
| miR-380-3p | -1.50154 | 0.00189 | 0.02853 |
| miR-301a-3p | -1.50235 | 0.00120 | 0.01910 |
| miR-639 | -1.511 | 0.00346 | 0.03892 |
| miR-200b-3p | -1.5338 | 0.00218 | 0.02958 |
| miR-1307-3p | -1.53851 | 0.00253 | 0.03237 |
| miR-4461 | -1.54493 | 0.00153 | 0.02341 |
| miR-3182 | -1.54772 | 0.00437 | 0.04424 |
| miR-1296-3p | -1.55054 | 0.00384 | 0.04090 |
| miR-487b-5p | -1.56445 | 0.00280 | 0.03488 |
| miR-532-3p | -1.57415 | 0.00305 | 0.03682 |
| miR-1287-5p | -1.5777 | 0.00291 | 0.03567 |
| miR-1-5p | -1.58736 | 0.00017 | 0.00406 |
| miR-27b-3p | -1.60098 | 2.54e-6 | 0.00016 |
| miR-769-3p | -1.60753 | 0.00222 | 0.02958 |
| miR-3614-5p | -1.64671 | 0.00197 | 0.02858 |
| miR-548g-3p | -1.69338 | 0.00018 | 0.00420 |
| miR-18b-5p | -1.69661 | 7.64e-5 | 0.00200 |
| miR-342-5p | -1.69872 | 3.68e-6 | 0.00021 |
| miR-130a-3p | -1.7146 | 0.00221 | 0.02958 |
| miR-1293 | -1.72002 | 0.00409 | 0.04289 |
| miR-1178-3p | -1.72188 | 0.00195 | 0.02858 |
| miR-758-3p+miR-411-3p | -1.73815 | 0.00039 | 0.00748 |
| miR-421 | -1.75132 | 0.00077 | 0.01332 |
| miR-582-5p | -1.76042 | 2.49e-5 | 0.00111 |
| miR-203a-5p | -1.77097 | 0.00374 | 0.04075 |
| miR-941 | -1.79728 | 0.00022 | 0.00472 |
| miR-1283 | -1.80312 | 0.00041 | 0.00757 |
| miR-521 | -1.80768 | 0.00025 | 0.00521 |
| miR-1910-3p | -1.82077 | 6.62e-5 | 0.00182 |
| miR-566 | -1.83869 | 0.00328 | 0.03850 |
| miR-455-5p | -1.83984 | 0.00022 | 0.00472 |
| miR-525-3p | -1.85453 | 0.00252 | 0.03237 |
| miR-526a+miR-518c-5p+miR-518d-5p | -1.87537 | 6.39e-6 | 0.00034 |
| miR-23c | -1.8786 | 5.36e-5 | 0.00165 |
| miR-378d | -1.87911 | 3.82e-5 | 0.00132 |
| miR-299-3p | -1.88191 | 3.51e-5 | 0.00132 |
| miR-196a-3p | -1.89448 | 5.71e-5 | 0.00169 |
| miR-675-5p | -1.89526 | 3.79e-5 | 0.00132 |
| miR-1266-5p | -1.89699 | 7.83e-5 | 0.00200 |
| miR-767-5p | -1.90447 | 0.00314 | 0.03739 |
| miR-1910-5p | -1.90965 | 0.00217 | 0.02958 |
| miR-210-3p | -1.91076 | 3.30e-5 | 0.00132 |
| miR-3074-3p | -1.93382 | 1.99e-5 | 0.00093 |
| miR-320b | -1.96685 | 0.00028 | 0.00554 |
| miR-891a-5p | -1.97181 | 0.00013 | 0.00308 |
| miR-10a-5p | -1.97345 | 0.00064 | 0.01157 |
| miR-4707-5p | -1.99504 | 3.32e-5 | 0.00132 |
| miR-1254 | -2.00992 | 0.00110 | 0.01824 |
| miR-424-5p | -2.01427 | 0.00112 | 0.01832 |
| miR-663a | -2.03251 | 0.00342 | 0.03892 |
| miR-22-3p | -2.11083 | 4.17e-12 | 3.33e-9 |
| miR-654-5p | -2.13424 | 0.00072 | 0.01279 |
| miR-378e | -2.14752 | 1.72e-6 | 0.00011 |
| miR-1247-5p | -2.20056 | 8.27e-7 | 6.00e-5 |
| miR-5196-5p | -2.21596 | 3.92e-7 | 4.47e-5 |
| miR-524-3p | -2.21834 | 6.11e-5 | 0.00174 |
| miR-6721-5p | -2.22367 | 0.00029 | 0.00561 |
| miR-489-3p | -2.24 | 4.87e-8 | 6.47e-6 |
| miR-1306-5p | -2.24311 | 0.00212 | 0.02958 |
| miR-30a-3p | -2.25435 | 6.55e-7 | 5.36e-5 |
| miR-3130-3p | -2.31145 | 4.30e-5 | 0.00143 |
| miR-516a-5p | -2.3301 | 4.94e-5 | 0.00158 |
| miR-4521 | -2.65689 | 0.00021 | 0.00457 |
| miR-873-3p | -3.02759 | 5.29e-7 | 5.27e-5 |
| miR-590-3p | -3.03772 | 1.08e-8 | 1.72e-6 |
| miR-1269a | -3.45008 | 6.72e-7 | 5.36e-5 |
| miR-1972 | -4.98502 | 1.33e-10 | 4.89e-8 |
| miR-4454+miR-7975 | -7.70364 | 1.84e-10 | 4.89e-8 |

**Supplementary table 8 Normalized miRNA Expression (relative to RNU6B)**

| Sample | **miR-100-5p** | **miR-150-5p** | **miR-223-3p** | **miR-494-3p** | Group |
| --- | --- | --- | --- | --- | --- |
| **TC-01** | 2,65 | 20,58 | 52,73 | 27,7 | B-Ly |
| **TC-02** | 0,2 | 2,98 | 1,78 | 103,04 | B-Ly |
| **TC-03** | 0,81 | 9,53 | 4,86 | 14,68 | B-Ly |
| **TC-04** | 2,6 | 78,37 | 11,44 | 632,37 | B-Ly |
| **TC-05** | 1,18 | 17,48 | 10,32 | 41,44 | B-Ly |
| **TC-06** | 4,09 | 122,12 | 3732,21 | 127,97 | B-Ly |
| **TC-07** | 0,28 | 3,33 | 4,4 | 15,35 | B-Ly |
| **TC-08** | 0,31 | 3,5 | 4,38 | 12,87 | B-Ly |
| **TC-09** | 1,59 | 50,32 | 69,57 | 46,57 | B-Ly |
| **TC-10** | 1,82 | 15,1 | 12,68 | 8,62 | B-Ly |
| **TC-11** | 8,85 | 927,31 | 169,94 | 2278,42 | B-Ly |
| **TC-12** | 1,32 | 19,77 | 11,71 | 165,46 | B-Ly |
| **TC-13** | 6,95 | 46,57 | 17,62 | 388,9 | B-Ly |
| **TC-15** | 2,37 | 81,73 | 33,2 | 21,27 | B-Ly |
| **TC-17** | 1,78 | 58,96 | 14,97 | 74,74 | B-Ly |
| **TC-18** | 1,83 | 87,44 | 64,23 | 54,46 | B-Ly |
| **TC-19** | 1,66 | 124,77 | 39,57 | 232,91 | B-Ly |
| **TC-20** | 2,14 | 5,66 | 12,32 | 429,48 | B-Ly |
| **TC-21** | 2,73 | 14,57 | 4,29 | 56,08 | B-Ly |
| **TC-23** | 2,39 | 8,26 | 7,97 | 97,1 | B-Ly |
| **TC-24** | 1,48 | 23 | 24,01 | 132,07 | B-Ly |
| **TC-52** | 1,04 | 10,06 | 6,31 | 20,62 | B-Ly |
| **TC-55** | 3,64 | 14,84 | 29,85 | 12,88 | B-Ly |
| **TC-60** | 0,97 | 143,34 | 1,2 | 97,34 | B-Ly |
| **TC-61** | 2,04 | 8,31 | 6,48 | 76,38 | B-Ly |
| **TC-62** | 0,45 | 6,79 | 2,29 | 32,9 | B-Ly |
| **TC-39** | 0,06 | 79,66 | 201,24 | 3,46 | B-AL |
| **TC-41** | 0,35 | 387,13 | 759,15 | 7,53 | B-AL |
| **TC-43** | 0,45 | 987,84 | 4958,54 | 6,95 | B-AL |
| **TC-44** | 0,15 | 126,03 | 629,39 | 0,76 | B-AL |
| **TC-45** | 0,37 | 289,85 | 3465,21 | 4 | B-AL |
| **TC-46** | 3,7 | 1279,73 | 1195,26 | 7,57 | B-AL |
| **TC-49** | 0,17 | 118,19 | 1738,5 | 8,14 | B-AL |
| **TC-76** | 2,11 | 4511,83 | 1567,74 | 4,2 | LN (normal control) |
| **TC-77** | 2,77 | 2518,77 | 236,74 | 3,78 | Tonsil (normal control) |
| **TC-78** | 1,9 | 1743,18 | 241,63 | 5,06 | Tonsil (normal control) |
| **TC-80** | 2,17 | 3650,95 | 1059,84 | 7,02 | Mix TC 76 and TC 78 |
| **TC-81** | 2,15 | 3049,72 | 511,54 | 4,92 | Mix TC 76, TC 77 and TC 78 |
| **B-AL vs B- Ly** |  |  |  |  |  |
| u-Mann Whitney | p=0.021 | p=0.000753 | p=0.000195 | p=0.000074 |  |
| Rosalind ® | p-adj 7.66 e-12 | p-adj 1.25 e-4 | p-adj 3.84 e-4 | p-adj 7.45 e-5 |  |
| **B-AL vs NCLT** |  |  |  |  |  |
| u-Mann Whitney | 0.033758 | 0.002525 | 0.267677 | 0.679942 |  |
| Rosalind® | p-adj 0.09 | p-adj 0.04183 | p-adj 0.07 | p-adj 0.15 |  |
| **B-Ly vs NCLT** |  |  |  |  |  |
| u-Mann Whitney | p=0.286143 | p=0.000530 | p=0.001373 | p=0.000530 |  |
| Rosalind® | p-adj 0.84 | p-adj 5.58 e-6 | p-adj 0.01267 | p-adj 0.01343 |  |
